# Unicystic Ameloblastoma with Mural Proliferation Managed by Conservative Treatment

**DOI:** 10.1155/2016/3089540

**Published:** 2016-08-17

**Authors:** Natália Galvão Garcia, Denise Tostes Oliveira, Moacyr Tadeu Vicente Rodrigues

**Affiliations:** ^1^Department of Stomatology, Area of Pathology, Bauru School of Dentistry, University of São Paulo, 17012-901 Bauru, SP, Brazil; ^2^Department of Stomatology, Sao Lucas School, 76805-846 Porto Velho, RO, Brazil

## Abstract

Unicystic ameloblastoma is a distinguishable entity of ameloblastomas, characterized by slow growth and being relatively locally aggressive. Three histological types are recognized according to the degree of ameloblastomatous epithelial extension, namely, luminal, intraluminal, and mural types. This classification has a direct bearing on their biological behavior, treatment, and prognosis. However, there is difficulty in determining the most appropriate form of treatment for unicystic ameloblastoma. We present a case of unicystic ameloblastoma that occurred in the right posterior mandible of 19-year-old girl, which was enucleated and did not recur after 12-month follow-up.

## 1. Introduction

Ameloblastomas are the most common form of aggressive benign tumors of the jaws [1]. The ameloblastomas are classified into several clinic-radiographic and histological types [2]. In the 2005 World Health Organization classification the ameloblastoma is divided into (1) solid/multicystic, (2) extraosseous/peripheral, (3) desmoplastic, and (4) unicystic [3]. Unicystic ameloblastoma is a distinguishable entity of ameloblastomas, characterized by slow growth and being relatively locally aggressive [4]. Three histological types are recognized according to the degree of ameloblastomatous epithelial extension, namely, luminal, intraluminal, and mural types [1–4]. This classification has a direct bearing on their biological behavior, treatment, and prognosis. However, there is difficulty in determining the most appropriate form of treatment for unicystic ameloblastoma [1]. We present case of unicystic ameloblastoma that occurred in the right posterior mandible of 19-year-old girl. The lesion was enucleated, and no recurrence was detected after 12-month follow-up.

## 2. Case Report

**Figure 1:**
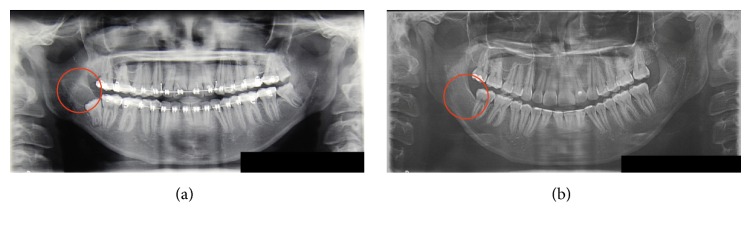
Panoramic radiograph before and after treatment. (a) Panoramic radiograph showed that the presence of a unilocular radiolucent lesion was shown in the right mandibular ramus, involving the impacted tooth 48. (b) The patient was followed up and, 12 months later, no sign of recurrence was detected.

A 19-year-old girl was undergoing orthodontic treatment, and the panoramic radiograph showed the presence of a unilocular radiolucent lesion in the right mandibular ramus, involving the impacted tooth 48 (Figure 1(a)). There was no associated pain and difficulty in opening the mouth, chewing, or articulating. The oral mucosa was normal and there was no expansion of the cortical bone.

The clinical diagnosis was a dentigerous cyst and the patient underwent enucleation of the lesion. During surgery, the cystic lesion, which enclosed a permanent lower first molar, was easily separated from the surrounding bone since it had an evident capsule. Tooth 48 was also extracted. The entire specimen was then submitted for histopathologic examination.

**Figure 2:**
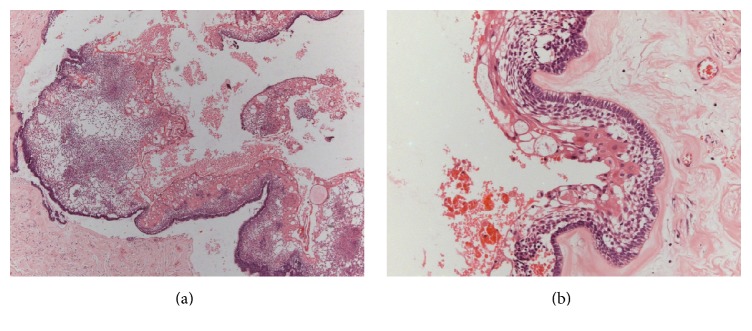
Microscopic characteristics. ((a) and (b)) A cystic cavity lined with epithelium was observed, in which the basal cells were columnar, hyperchromatic, and palisaded and had reversed polarity (Hematoxylin and Eosin: (a) 25x; (b) 200x).

**Figure 3:**
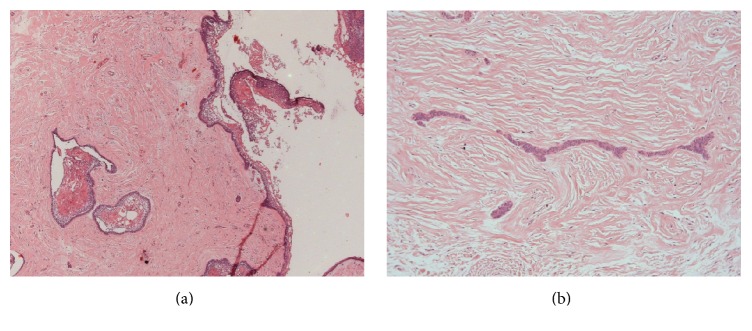
Microscopic characteristics. ((a) and (b)) Underlying the fibrous capsule, proliferation of neoplastic cells was noted, sometimes arranged in strands and sometimes in islands, with areas of squamous metaplasia, in addition to several islands of odontogenic epithelium (Hematoxylin and Eosin: (a) 25x; (b) 100x).

Microscopically, cystic cavity lined by epithelium was observed in which the basal cells were columnar, hyperchromatic, and palisaded and with reverse polarity (Figures 2(a) and 2(b)). In some areas epithelial proliferation into the lumen was observed with some cells resembling the stellate reticulum and foci of squamous metaplasia. Underlying the fibrous capsule proliferation of neoplastic cells was noted sometimes arranged in strands and sometimes in islands, with areas of squamous metaplasia, besides several islands of odontogenic epithelium (Figures 3(a) and 3(b)). The final diagnosis established based on the association of clinical and microscopic features was of unicystic ameloblastoma with mural proliferation.

The patient was followed up and 12-month later no sign of recurrence was detected (Figure 1(b)).

## 3. Discussion

Unicystic ameloblastoma is a rare variant of ameloblastoma that was first described by Robinson and Martinez in 1977, referring to those cystic lesions that show clinical and radiologic characteristics of an odontogenic cyst but in histological examination it shows a typical ameloblastomatous epithelium lining part of the cyst cavity, with or without luminal and/or mural tumor proliferation [4, 5].

Based on the character and extent of tumor cell proliferation within the cyst wall, several histologic subtypes of unicystic ameloblastoma are recognized, which include those of simple cystic nature, those with intraluminal proliferation nodules, and those containing infiltrative tumor islands in the cyst walls [6, 7].

According to Philipsen and Reichart, the first two groups of lesions may be treated successfully by enucleation or curettage; it has been suggested that recurrence following conservative surgery is more likely to occur in the third group and that these lesions should therefore be treated by radical resection, as for a solid or multicystic ameloblastoma [8].

However, there is difficulty in determining the most appropriate form of treatment for these lesions, with the treatment of unicystic ameloblastoma being still very controversial [1, 9, 10].

This happens due to the misapplication of the term “mural” [7]. According to some authors, the presence of mural proliferation increases the rate of recurrence defining the best and appropriate surgical treatment for this lesion [5–8]. For others, the choice of treatment for unicystic ameloblastoma, enucleation or surgical resection, depends on the severity and type of odontogenic epithelial mural proliferation [6, 7].

The term “mural” describes the extent to which amelobastomatous changes penetrate the connective tissue layer of a cyst. Just as a mural painting covers only the surface of a wall, a mural ameloblastoma does not penetrate the epithelial lining of a cyst [7, 11, 12]. Due to the misapplication of the classification, statistics concerning recurrence rates of such so-called ameloblastomas related to the use of specific treatment approaches have been inaccurate and in some cases dangerously misleading [7].

For Marx and Stern, ameloblastoma “in situ” developing in and limited to the epithelial lining of a cyst and also ameloblastoma “microinvasive” arising from the epithelial lining and proliferating into the connective tissue layer of the cyst should be treated with enucleation. Yet, ameloblastoma “invasive” arising from the epithelial lining and proliferation through the complete thickness of the connective tissue layer of a cyst should be treated with resection [7].

In the present case, microscopically epithelial proliferation was observed into the lumen and into the connective tissue layer of the cyst. The final diagnosis established was of unicystic ameloblastoma with mural proliferation. Based on this diagnosis, the treatment recommended, for most authors, is the surgical resection. However, according to Marx and Stern, our unicystic ameloblastoma is “microinvasively” arising from the epithelial lining and proliferating into the connective tissue layer of the cyst, where the enucleation is considered one viable treatment.

Besides, the age of the patient and clinical and radiologic features were considered; thus the enucleation was the treatment of choice. The patient was followed up and one year later there was no sign of recurrence.

In literature, the recurrence after conservative treatment of unicystic ameloblastoma is reported to be between 10 and 25% but these reports do not specify the histologic subtypes of the primary lesion. Due to this, many professionals choose resection as treatment, which most often is unnecessary [6, 13, 14].

Therefore, in addition to analyzing the clinical and radiological data of the lesion, histopathological examination is of great importance for an accurate diagnosis and consequently to choose the most appropriate treatment.
